# CRISPR Toolbox for Genome Editing in *Dictyostelium*

**DOI:** 10.3389/fcell.2021.721630

**Published:** 2021-08-18

**Authors:** Kensuke Yamashita, Hoshie Iriki, Yoichiro Kamimura, Tetsuya Muramoto

**Affiliations:** ^1^Department of Biology, Faculty of Science, Toho University, Funabashi, Japan; ^2^Laboratory for Cell Signaling Dynamics, RIKEN, Center for Biosystems Dynamics Research (BDR), Osaka, Japan

**Keywords:** *Dictyostelium*, biomedical model, CRISPR/Cas9, Cas9-NG, SpRY, gene manipulation, CRISPRi, drug-inducible knockout

## Abstract

The development of new techniques to create gene knockouts and knock-ins is essential for successful investigation of gene functions and elucidation of the causes of diseases and their associated fundamental cellular processes. In the biomedical model organism *Dictyostelium discoideum*, the methodology for gene targeting with homologous recombination to generate mutants is well-established. Recently, we have applied CRISPR/Cas9-mediated approaches in *Dictyostelium*, allowing the rapid generation of mutants by transiently expressing sgRNA and Cas9 using an all-in-one vector. CRISPR/Cas9 techniques not only provide an alternative to homologous recombination-based gene knockouts but also enable the creation of mutants that were technically unfeasible previously. Herein, we provide a detailed protocol for the CRISPR/Cas9-based method in *Dictyostelium*. We also describe new tools, including double knockouts using a single CRISPR vector, drug-inducible knockouts, and gene knockdown using CRISPR interference (CRISPRi). We demonstrate the use of these tools for some candidate genes. Our data indicate that more suitable mutants can be rapidly generated using CRISPR/Cas9-based techniques to study gene function in *Dictyostelium*.

## Introduction

Many human diseases, such as cancer, heart disease, and diabetes, are characterized by multifactorial genetic inheritance (Khera et al., 2018; Ishigaki et al., 2020). Therefore, manipulating the genome is crucial for elucidating the causes of disease and systematically studying the many genes that underlie intracellular processes. To understand gene function, many researchers edit specific DNA sequences in the genome. For instance, homologous recombination in ES cells introduces mutations in tumor suppressor genes and oncogenes involved in the onset and progression of the multiple diseases (Thomas et al., 1986; Mansour et al., 1988; Di Cristofano et al., 1998; Capecchi, 2005). Various methods to generate mutations, including knockouts, knock-ins, large deletions and point mutations, have been developed using homologous recombination; however, the techniques are limited by their inefficiency and time requirements (Gerlai, 2016). Recent advances in the development of CRISPR/Cas9 have made it possible to program specific DNA cleavage in eukaryotic cells precisely and efficiently. By simply expressing Cas9 with a sgRNA that is complementary to a target sequence, it is possible to introduce any genetic modification effectively at nearly any position within the genome and minimize off-target effects (Jinek et al., 2012; Cong et al., 2013). In the last decade, this useful genetic tool has greatly improved our understanding of the onset and progression of human genetic diseases.

Research on genes associated with human diseases have also been conducted using the biomedical model organism *Dictyostelium* (Williams et al., 2006; Martin-Gonzalez et al., 2021). This organism lacks the complexity of the metazoan models *Caenorhabditis elegans* and *Drosophila melanogaster*, but it shares many fundamental biological processes related to human diseases such as mitochondrial dysfunction, lysosomal dysregulation, and autophagy dysfunction. In particular, biological processes including cell motility and cytoskeletal rearrangement, cell shape regulation, cell cycle control, DNA repair mechanisms, phagocytosis, and pinocytosis are actively studied (Kessin, 2001; Eichinger et al., 2005). Genome sequence data revealed that many genes related to human disease are present in *Dictyostelium* and various orthologs have already been characterized to elucidate their functional similarity (Pears and Lakin, 2014; Mesquita et al., 2017; Huber and Mathavarajah, 2019; Vogel et al., 2019). Because this organism is haploid, it is easy to generate genetic mutants, and phenotypes can be directly determined in the clone without further manipulation. A wide range of genetic techniques are available including homologous recombination–based methods (knockout, knock-in, and point mutation generation) and protein overexpression and expression of fusion-tagged proteins using various expression vectors (Gaudet et al., 2007; Veltman et al., 2009a). Genome editing using CRISPR/Cas9 has also been added to the toolbox for functional analysis in *Dictyostelium*, making it possible to modify genomes with higher efficiency than methods based on homologous recombination (Sekine et al., 2018; Iriki et al., 2019; Asano et al., 2021).

Since the first demonstrations of programmed DNA cleavage by Cas9 nuclease from *Streptococcus pyogenes* (SpCas9) (Jinek et al., 2012), the discovery and engineering of CRISPR/Cas9 systems as tools for genome manipulation has progressed rapidly. In this paper, we focus on CRISPR/Cas9-based technologies to create knockouts, knock-ins, point mutations and deletion mutants. CRISPR applications such as epigenetic modification, chromatin manipulation, and live cell chromatin imaging are expanding and valuable for functional analyses (Adli, 2018; Knight et al., 2018; Pickar-Oliver and Gersbach, 2019; Anzalone et al., 2020), but will not be dealt with in this article. The Cas9 nuclease is guided by a sgRNA for target-site recognition and then generates a DNA double-strand break (DSB) using two nuclease domains (RuvC and HNH) (Cong et al., 2013; Mali et al., 2013; Ran et al., 2013). A blunt-ended DSB typically occurs at the site preceding the three nucleotides upstream of a protospacer adjacent motif (PAM), followed by DSB repair through an end-joining DNA repair pathway. Uncontrollable, but considered predictable, insertion/deletion (indel) mutations can typically be achieved through non-homologous end joining (NHEJ) or microhomology-mediated end joining (MMEJ); hence, most knockout mutants have been generated by frameshift mutations through this process. If the targeted gene is essential, mutants carry only non-frameshift mutations (You et al., 2020). In the presence of a donor DNA template, homology directed repair (HDR) occurs and the large tagged DNA sequence is knocked-in. By contrast, in the presence of single-stranded DNA oligonucleotide (ssODN) donors, precise point mutations or single nucleotide substitutions are introduced into a target site in the genome. Recently, newly engineered SpCas9 and Cas9 orthologs have been discovered, resulting in an expansion in the targeting scope of the genome (Ran et al., 2015; Kim et al., 2017; Chatterjee et al., 2018). SpCas9 recognizes the PAM “NGG”, which is located 3′-end of the target sequence. Different versions of Cas9, such as xCas9, SpCas9-NG and SpRY, recognize a broader variety of PAM recognition sequences with less stringent motif requirements (Hu et al., 2018; Nishimasu et al., 2018; Walton et al., 2020).

Using these various types of Cas9, a wide range of genome editing applications, including knockouts, inducible knockouts, knockdowns, knock-ins, point mutations and deletions, have been established in *Dictyostelium* (Table 1; Muramoto et al., 2019; Asano et al., 2021). SpCas9 is commonly used to create knockouts and has the highest efficiency among the available vectors, but the system is limited to applications in which genome editing does not need to be controlled temporally. In this study, we developed a doxycycline-inducible CRISPR/Cas9 system that allows for temporal control of genome editing activity. SpCas9 is capable of editing the target sequence in the presence of canonical “NGG” PAMs; however, due to the presence of AT-enriched regions in *Dictyostelium* genome, design of appropriate targets is elusive. To overcome this limitation, the newly engineered SpCas9 variants SpCas9-NG and SpRY were developed and are able to recognize a wider range of PAM sequences (Asano et al., 2021). This system provides valuable tools that will significantly expand the number of targetable gene loci available to generate mutants, even in AT-rich regions, with sufficient efficiency. Cas9 nuclease sometimes cleaves off-target sites that possesses high sequence homology to the target sites. To reduce the probability of off-target effects and increase specificity for DSB, a pair of sgRNAs for a Cas9 nickase were used. Inactivation of either of the nuclease domains generates a Cas9 nickase, which creates a nick on one strand of DNA, and precise gene knockouts and long deletions were achieved (Iriki et al., 2019). Moreover, a mutation in both nuclease domains generates a catalytically inactive Cas9 (dCas9), which is still able to bind to specific DNA sequences. dCas9 is a useful tool for knockdown (CRISPRi) (Gilbert et al., 2013; Qi et al., 2013). In this study, we developed a straightforward CRISPRi system to reduce mRNA and protein levels in the cells.

**TABLE 1:** Comparison of various Cas9 nucleases and applications in genome editing.

Cas9 nucleases	PAM	Key features	Applications
SpCas9	NGG	Double-strand break Commonly used in genome editing	Knockout
SpCas9 (Dox-On)	NGG	Controllable genome editing Possibility of minimizing off-targets	Inducible knockout
SpCas9-NG	NG	Genome editing at NG PAM sequences Majority of genomic region editable	Knockout Knock-in (with donor DNA) Point mutation (with a ssODN)
SpRY	NR	Genome editing at NR PAM sequences (R = A and G) Majority of genomic region editable	Knockout Knock-in (with donor DNA) Point mutation (with a ssODN)
Cas9 nickase	NGG	Single-strand break Lower off-targets than Cas9	Large deletion Knock-in (with donor DNA) Point mutation (with a ssODN)
SpCas9-NG nickase	NG	Single-strand break at NG PAM sequences Lower off-targets than Cas9	Large deletion
dCas9	NGG	Lack of endonuclease activity Inhibition of gene expression	Knockdown (CRISPRi)
dCas9 (Dox-On)	NGG	Controllable inhibition of gene expression	Knockdown (CRISPRi)

In this methodological paper, we summarize these applications and focus on detailing the selection of appropriate CRISPR/Cas9 vectors and procedures for each technology to manipulate the *Dictyostelium* genome for the study of human disease-related genes.

## Materials and Equipment

### Reagents for Cell Culture

(1) *D. discoideum* cells (e.g., AX2, AX3 or any other cell line of interest, available from the Dicty Stock Center or NBRP Nenkin).
(2) *Klebsiella pneumoniae* KpGe strain (Lima et al., 2018) (Genome was sequenced and non-pathogenic strain, available from the Dicty Stock Center or NBRP Nenkin).
(3) HL5 medium including glucose (Formedium, HLG0102). Autoclave and store at RT.
(4) SM agar (Formedium, SMA0102). Sterilized by autoclaving and approximately 35 mL are poured into the 10 cm petri dishes. Store at 4°C.
(5) KK2 buffer: 16.5 mM KH_2_PO_4_ and 3.8 mM K_2_HPO_4_. Autoclave and store at RT.
(6) H50 buffer: 50 mM KCl, 20mM HEPES pH 7.0, 10 mM NaCl, 5 mM NaHCO_3_, 1mM NaH_2_PO_4_, 1 mM MgSO_4_. Sterilize and store at 4°C.
(7) Streptomycin stock solution: 50 mg/mL in distillated water, sterile filtered. Store at 4°C or freeze in aliquots.
(8) Blasticidin S stock solution (1,000×): 10 mg/mL in KK2, sterile filtered. Store at 4°C or freeze in aliquots.
(9) G418 stock solution: 20 mg/mL in KK2, sterile filtered. Store at 4°C or freeze in aliquots.
(10) Hygromycin B stock solution: 50 mg/mL in distillated water, sterile filtered. Store at 4°C or freeze in aliquots.
(11) Doxycycline stock solution: 10 mg/mL in distillated water, sterile filtered. Store at 4°C or freeze in aliquots.

### Reagents for Molecular Cloning

(1) Competent *E. coli* cells (e.g., TOP10 or any other suitable strain).
(2) LB broth Lennox (Formedium, LBX0102). Autoclave and store at RT.
(3) Ampicillin stock solution (1,000×): 100 mg/mL in distillated water, sterile filtered. Store at 4°C or freeze in aliquots.
(4) LB plates: 15.0 g of Agar, 20.0 g of LB broth Lennox, bring to 1 L and autoclave, cool to approximately 50°C, and add 1 ml of ampicillin stock solution.
(5) CRISPR/Cas9 all-in-one vectors. List of vectors is available in Table 2. (Available from NBRP Nenkin).

**TABLE 2:** CRISPR/Cas9 vectors.

CRISPR vector	Cas9 (* GFP fusion)	Inducible Expression	Number of tRNA–sgRNA	GG enzyme	Drug resistance	Transient / Stable	References
pTM1285	Cas9 *	–	1	BpiI	Neo	Transient	Sekine et al., 2018
pTM1599	Cas9	–	1	BpiI	Neo	Transient	Asano et al., 2021
pTM1416	Cas9 *	–	1	Esp3I	Neo	Transient	Asano et al., 2021
pTM1644	Cas9	–	1	Esp3I	Neo	Transient	Asano et al., 2021
pTM1756	Cas9	–	1	Esp3I	Hyg	Transient	This study
pTM1725	Cas9 *	–	2	BpiI	Neo	Transient	This study
pTM1859	Cas9	–	2	Esp3I	Neo	Transient	This study
pTM1860	Cas9	–	2	Esp3I	Hyg	Transient	This study
pTM1676	Cas9	Tet-On	1	Esp3I	Blast	Stable	This study
pTM1670	Cas9	Tet-On	1	Esp3I	Neo	Stable	This study
pTM1544	Cas9 nickase *	–	2	BpiI	Neo	Transient	Iriki et al., 2019
pTM1866	Cas9 nickase *	–	2	BpiI	Hyg	Transient	This study
pTM1702	dCas9	–	1	Esp3I	Neo	Stable	This study
pTM1869	dCas9	–	1	Esp3I	Hyg	Stable	This study
pTM1765	dCas9	–	2	Esp3I	Neo	Stable	This study
pTM1870	dCas9	–	2	Esp3I	Hyg	Stable	This study
pTM1826	dCas9	Tet-On	1	Esp3I	Blast	Stable	This study
pTM1827	dCas9	Tet-On	1	Esp3I	Neo	Stable	This study
pTM1718	Cas9-NG	–	1	BpiI	Neo	Transient	Asano et al., 2021
pTM1719	Cas9-NG	–	1	Esp3I	Neo	Transient	Asano et al., 2021
pTM1631	Cas9-NG	–	2	BpiI	Neo	Transient	This study
pTM1726	Cas9-NG nickase	–	2	BpiI	Neo	Transient	This study
pTM1668	SpRY	–	1	Esp3I	Neo	Transient	Asano et al., 2021
pTM1825	SpRY	–	1	Esp3I	Hyg	Transient	This study
pTM1748	SpRY	–	2	Esp3I	Neo	Transient	This study
pTM1865	SpRY	–	2	Esp3I	Hyg	Transient	This study

(6) Oligonucleotides for sgRNA construction. Detailed of construction methods are available in Procedure section.
(7) Annealing buffer (10×): 400 mM Tris-HCl pH 8.0, 200 mM MgCl_2_, 500 mM NaCl. Store at −20°C.
(8) T4 Polynucleotide kinase, 10 U/μL (Takara Bio, 2021A).
(9) T4 DNA ligase, 400 U/μL (NEB, M0202S).
(10) 10 × T4 DNA ligase buffer (NEB, B0202S).
(11) BpiI, 10 U/μL (Thermo Scientific, ER1011).
(12) 10 × Buffer G (Thermo Scientific, BG5).
(13) Esp3I, 10 U/μL (NEB, R0734S).
(14) 10 × CutSmart Buffer (NEB, B7204S).
(15) OneTaq or other equivalent Taq DNA polymerase (NEB, M0480S).
(16) Agarose (No particular preference).
(17) Plasmid DNA mini kit (No particular preference, we used FastGene Plasmid Mini Kit.).
(18) Plasmid DNA midi kit (No particular preference, we used NucleoBond Xtra Midi Kit.).
(19) Primer for PCR screening (Supplementary Table 1). Detailed of primer design is available in Procedure section.
(20) Primer for sequencing (Supplementary Table 1).

### Reagents for Mutation Detection Protocol

(1) DNA extraction buffer: 0.5 × Ex Taq buffer, 0.5% NP40, 50 ng/μL of Proteinase K. The buffer must be prepared fresh and kept on ice while using.
(2) KOD -Plus- Neo, 1 U/μL (TOYOBO, KOD-401).
(3) Ex Taq, 5 U/μL (Takara Bio, RR001A).
(4) Primer for PCR screening and sequencing (Supplementary Table 1).

### Equipment

(1) Cell culture incubator (set at 22°C).
(2) Sterile 10 cm culture dishes.
(3) Electroporator Xcell (BIO-RAD).
(4) Electroporation cuvettes: 1-mm gap.
(5) Thermal cycler.
(6) Microcentrifuge.
(7) DNA electrophoresis apparatus.
(8) Nanodrop (ThermoFisher).

### Software and Bioinformatic Tools

(1) Cas-Designer^1^ (Park et al., 2015).
(2) Cas-OFFinder^2^ (Bae et al., 2014).
(3) Standalone edition of Cas9-Designer to search with various PAM types.
(4) CRISPOR^3^ (Concordet and Haeussler, 2018).

## Stepwise Procedures

### Overview

**FIGURE 1:**
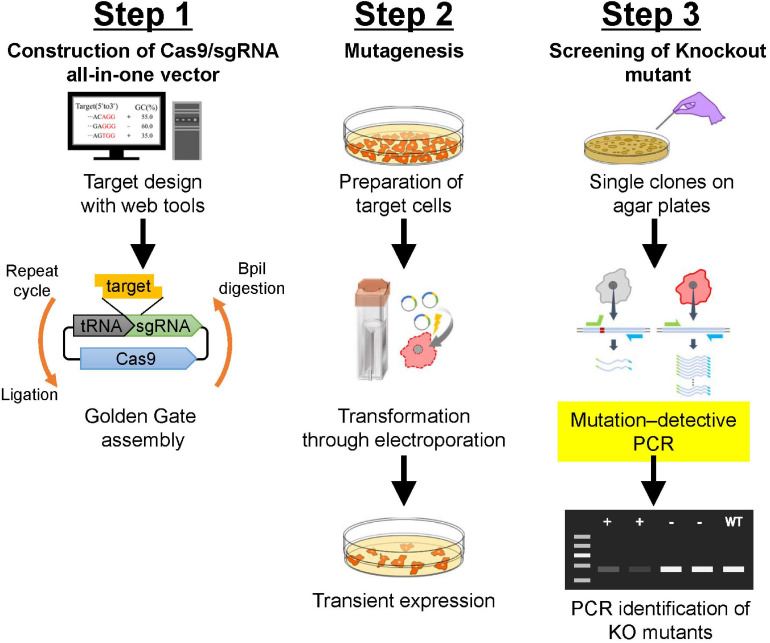
Schematic description of gene manipulation in *Dictyostelium* using CRISPR/Cas9. **Step 1**: Construction of CRISPR/Cas9 vector using Golden Gate assembly. Correctly integrated clones are selected using colony PCR followed by Sanger sequencing. **Step 2**: Transformation of *Dictyostelium* cells. Electroporation is used to transform *Dictyostelium*. Transient expression of CRISPR/Cas9 vector is induced via the addition of a small amount of antibiotics. **Step 3**: Screening of knock-out mutants. After isolation of individual colonies, mutation–detective PCR is conducted to identify positive knock-out mutants. For further validation of the mutation, genomic sequencing of the mutated region is analyzed.

Protocols described herein are an optimized version of the CRISPR/Cas9 system that uses co-expression of vectors for Cas9 and tRNA-flanked sgRNAs in *Dictyostelium*. These protocols include: selection of appropriate CRISPR/Cas9 vectors, identification of appropriate target sites in the genome, design of oligonucleotide-flanking overhang sequences for target cloning, Golden Gate cloning into a suitable CRISPR/Cas9 vector, transformation of the *Dictyostelium* cells, and screening and validation of mutants. A scheme for each of these protocols, from design of sgRNA to isolation of mutants, is shown in Figure 1.

### Selection of Appropriate CRISPR/Cas9 Vectors

Choosing the appropriate CRISPR/Cas9 vector is critical to the success of genome editing. The all-in-one vectors are easy-to-use genome-editing tools and consist of three modules: Cas9, tRNA–sgRNA, and a drug resistance gene (Table 2). The first module, Cas9 nuclease, has many options (Table 1); hence, we can select the appropriate vector according to the application in genome editing. Some of the vectors contain a GFP sequence followed by the Cas9 nuclease; however, genome editing can be induced with high efficiency regardless of the presence or absence of GFP. For temporal control of genome editing, doxycycline-inducible Cas9 vectors are available. The second module, tRNA–sgRNA, is an isoleucine tRNA and sgRNA cassette. A high level of sgRNA transcription is induced by an RNA polymerase III–dependent promoter for an isoleucine tRNA instead of the commonly used U6 promoter in various organisms. High levels of sgRNA readily form a ribonucleoprotein (RNP) complex with Cas9 and target specific genomic sequences. The number of tRNA–sgRNA cassettes required depends on the application in genome editing. When disrupting two genes at the same time, generating a deletion with a nickase, or inducing repression of gene expression are desired, a vector with two tRNA–sgRNA cassettes is useful. The cassette contains the Type IIS restriction enzyme site BpiI or Esp3I, which is used to insert the target sequence between the tRNA and sgRNA sequences via Golden Gate assembly. For the third module, the drug resistance gene, three antibiotic choices are available: neomycin, hygromycin, and blasticidin.

The three modules were cloned into pBlueScript II or pDM304-derived vectors to obtain transient or stable expression, respectively. Because pBlueScript II does not contain an element for extra-chromosomal replication in *Dictyostelium*, Cas9 nuclease is only expressed for a short period of time after electroporation, which minimizes off-target effects. Each vector contains one of the drug resistance genes used in *Dictyostelium*, and transient expression is induced when a low volume of the appropriate antibiotics is added. Moreover, it is better to select a vector with a hygromycin-resistance cassette than a vector with a G418-resistance cassette when genome editing of G418-resistant cells is desired. For the dCas9 or doxycycline-controlled inducible Cas9 system, pDM304-derived vectors (Veltman et al., 2009a) were used to obtain stable expression cell lines. These vectors have no or extremely low levels of off-target effects because of expression of dCas9, which lacks nuclease activity, or temporal expression of Cas9 nuclease, respectively. All of the plasmids and predicted sequences are available from the National BioResource Project “Cellular Slime Molds” (NBRP Nenkin, https://nenkin.nbrp.jp/locale/change?lang=en), which collects and distributes bioresources of *Dictyostelium* and other cellular slime molds that mainly originate in Japan.

### Design of Guide RNA for Gene Knockouts, Knockdowns, Knock-ins and Point Mutations

**FIGURE 2:**
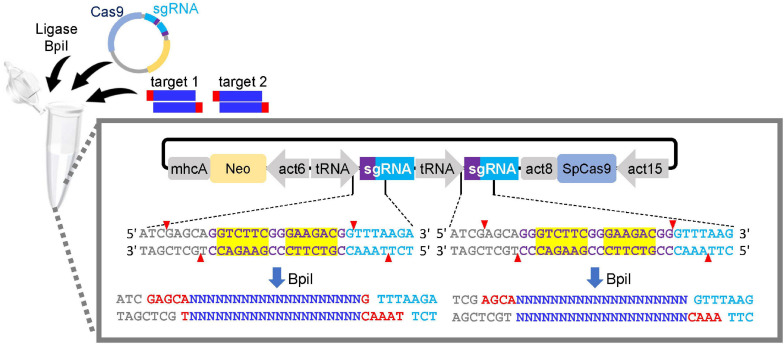
Single cloning step of a dual sgRNA expression vector. Annealed oligonucleotides are ligated to the tRNA–sgRNA junctions via Golden Gate reaction. DNA sequences of BpiI sites are indicated in yellow box. As the BpiI site is non-palindromic, re-digestion/ligation is not possible. Red and blue letters show the overhangs for cloning and target sequences of sgRNAs, respectively. CRISPR vector, T4 DNA ligase and annealed oligonucleotides for the first and second targets are mixed in a single tube to integrate two targets.

(1) Identify a gene or region of interest to introduce mutations.
  ***Note***: For knockouts, targets can be selected within the gene. The gene function can be completely disrupted by designing target sequences in the first half of the gene or in a functional domain, if possible. By contrast, the range of knock-ins and point mutations available is limited because the target can only be designed around the narrow genomic region of interest. Cas9 variants with relaxed PAM sequences, such as Cas9-NG and SpRY, are useful for generation of knock-ins and point mutations because they increase the number of places where targets can be designed. In knockdowns, the target is designed inside and upstream of the gene including the transcriptional initiation site.
(2) Find candidate target sequences in the region of interest using web tools such as Cas-Designer.
  ***Note***: 5′-NGG-3′ is a canonical PAM sequence that is recognized by SpCas9. The output is 23 nucleotides, which includes 20 nucleotides for the target and 3 nucleotides for the PAM. PAM sequences appear in either DNA strand (the upper strand or complementary lower strand). Four-thymidine repeats should be avoided because they are a termination signal for RNA polymerase III. More than 20% of GC content without the PAM sequence is recommended.
  An out-of-frame score of 66 or higher returned by Cas-designer is recommended, but even if the score is lower than 66, it is possible to edit a gene of interest. It is more important to select a target with a mismatch score of zero to prevent off-target cleavage. The potential for genome wide off-target effects remains undetermined in *Dictyostelium*, but a non-specific mutation was rarely observed when a target sequence with single-nucleotide mismatch in the 20-nucleotide target sequence was selected. Indeed, a single-nucleotide mismatch at the 3rd nucleotide from the NGG PAM prevented a non-specific mutation, while a target sequence with a mismatch at the 19th nucleotide from the PAM sometimes showed off-target effects. Because sequences with a single-nucleotide mismatch are not selected as targets, we expect the non-specific mutation rate to be low.
  Further consideration is needed to design the target. Designing targets around AT-rich regions or regions containing repeat sequences should be avoided to allow the design of good primers that amplify the locus to detect the mutation via DNA sequencing. Although the commonly used U6 promoter prefers a G at the 5′ end for effective expression, no extra G is necessary in our CRISPR/Cas9 vectors because of the tRNA-based expression system. In addition to Cas-Designer, the program CRISPOR is also available (Concordet and Haeussler, 2018).
(3) Synthesize two complementary oligonucleotides corresponding to the target sequence. Appropriate overhang sequences should be added for Golden Gate cloning.
  ***Note***: The 20 bp target is included in the oligonucleotides but the PAM sequence should not be added. Add 5′-AGCA-3′ to the 5′ end of the sense oligonucleotide and 5′-AAAC-3′ to the 5′ end of the antisense oligonucleotide for the Golden Gate digestion/ligation reaction. These overhangs are common to both BpiI and Esp3I mediated Golden Gate reactions. When cloning two sgRNAs into a vector to generate a double knockout, different overhangs are added to the oligonucleotides (Table 3). Two target sequences can be integrated into a CRISPR vector with a single tube reaction. Schematic illustration of the overhangs is shown in Figure 2.

**TABLE 3:** Appropriate overhang sequences for sgRNA oligonucleotides.

	Sequence (5′ to 3′)
First sgRNA site	
Sense oligonucleotide	AGCA-N_20_
Antisense oligonucleotide	AAAC-N_20_
Second sgRNA site	
Sense oligonucleotide	GAGCA-N_20_-G
Antisense oligonucleotide	TAAAC-N_20_-T

*N_20_ = 20 nucleotides genomic target.*

### Design of Guide RNA for Generation of Gene Deletions and Point Mutations Using Cas9 Nickase

**FIGURE 3:**
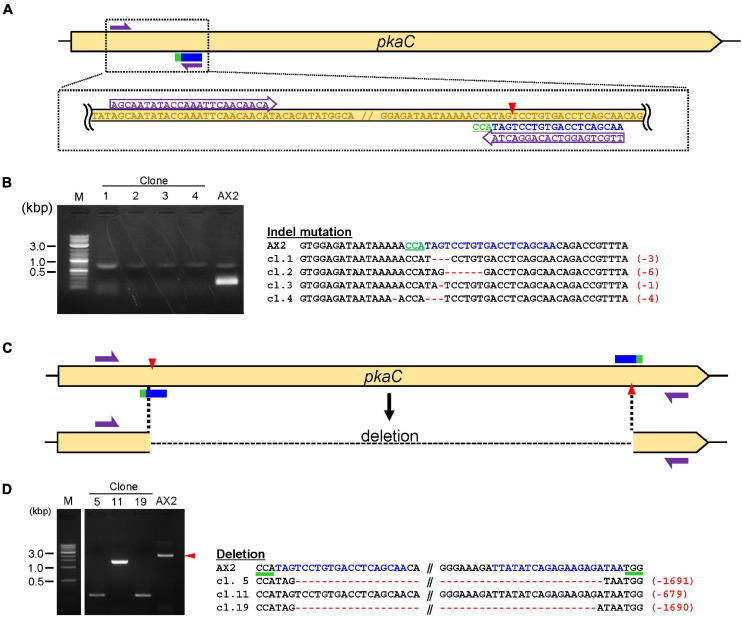
Validation of knock-out and deletion mediated by CRISPR/Cas9. **(A)** Schematic diagram of the target site for knock-out. Target sequences in *pkaC* and primers for mutation–detective PCR are shown. **(B)** Results of mutation–detective PCR and sequencing of the target regions are shown. The PCR products that revealed low amplification compared to the control were candidate mutants. The target sequence is in blue and mutations are in red. Numbers in parentheses indicate the number of modified nucleotides. **(C)** Schematic illustration of target sites for deletion mediated by Cas9-nickase. **(D)** PCR and Sequencing results of deletion mutants. The PCR fragments that were different in size compared to the control were deletion mutants.

(1) Identify a gene of interest to generate a deletion or point mutation.
(2) Find a pair of target sequences in the region using a web tool.
  ***Note***: A combination of sgRNAs with PAM sites flanking the opposite DNA strand should be selected in order to induce double-nicking (Figure 3). If the target sequence cannot be designed in the appropriate position, consider using Cas9-NG nickase, where NG is the PAM sequence.

(3) Synthesize sgRNA sequences as a pair of forward and reverse oligonucleotides. Each pair of oligonucleotides contains unique overhangs for Golden Gate assembly.
  ***Note***: The unique overhangs are the same as the ones used in double gene knockouts shown in Table 3. Details are also shown in Figure 2.

### Design of a HDR Template for a Tag Knock-in or Precise Point Mutation

By combining the CRISPR/Cas9 vector with ssODN or donor DNA, a tag knock-in or single nucleotide substitution can be efficiently achieved, respectively.

#### Design of ssODN for Precise Point Mutations

(1) The ssODN consists of substituted nucleotides flanked by homology arms on both left and right sides. The base substitutions are designed to be placed near the center of the ssODN and the targeting sgRNA region is located near the mutation. Homology arms are designed to include 30–80 perfectly matched nucleotides on both sides.
(2) Synthesize an ∼120 nucleotide ssODN as a template for HDR. Use 2.5 μl of 10 μM ssODN per electroporation.

#### Design of Donor DNA for Tag Knock-in

(1) To knock-in relatively longer sequences such as GFP, HDR template with the tag sequence is amplified using PCR with primers containing the homologous sequence of the target gene. The homologous sequences at both ends are designed to be 30–90 nucleotides in length, with 60 nucleotides generally being sufficient to achieve high efficiency. Shortening or extending the length of the homologous sequence alters knock-in efficiency (Asano et al., 2021).
(2) Amplify the tag sequence containing the homologous sequence using PCR with a high-fidelity DNA polymerase. Prepare ∼2 μg of donor DNA per electroporation.

### Molecular Cloning of CRISPR/Cas9 Constructs

#### Preparation of Annealed Oligonucleotides for the Target Gene

(1) Dissolve oligonucleotides to a concentration of 10 μM with distilled water.
(2) Prepare the following annealing mixture (10 μL):
  10 μM sense oligo for target 4.5 μL
  10 μM antisense oligo for target 4.5 μL
  10 × annealing buffer 1.0 μL
(3) Anneal the mixture in a thermal cycler under the following conditions:
  95°C for 5 min, followed by slowly cooling to 25°C (1°C/min).

#### Cloning of Annealed Oligonucleotides Into the tRNA–sgRNA Junction Using Golden Gate Assembly

(4) Set up a Golden Gate reaction under the following conditions.
<single tRNA–sgRNA expression vector (4.0 μL)>
  CRISPR/Cas9 vector (25 ng/μL) 0.8 μL
  10 × T4 DNA ligase buffer 0.4 μL
  T4 DNA ligase 0.2 μL
  Annealed oligo 0.3 μL
  BpiI^∗^ 0.2 μL
  Autoclaved milli-Q water 2.1 μL
<dual tRNA–sgRNA expression vector (8.0 μL)>
  CRISPR/Cas9 vector (25 ng/μL) 1.6 μL
  10 × T4 DNA ligase buffer 0.8 μL
  T4 DNA ligase 0.4 μL
  Annealed oligo first target 0.3 μL
  Annealed oligo second target 0.3 μL
  BpiI^∗^ 0.4 μL
  Autoclaved milli-Q water 4.2 μL
  ***Note***: ^∗^If there is an Esp3I site between tRNA and sgRNA, Esp3I is used instead of BpiI. When using Esp3I, adding CutSmart buffer to the reaction mixture may increase the efficiency of the Golden Gate reaction.
(5) Digestion/ligation reaction is conducted under the following thermocycling conditions:
  37°C for 5 min
  16°C for 15 min
  Repeat 5–8 times
(6) Prepare complete digestion reaction to prevent contamination of the non-integrated vector by combining:
<single tRNA–sgRNA expression vector (4.0 μL)>
  Golden Gate product 4.0 μL
  10 × Buffer G^∗^ 0.4 μL
  BpiI 0.2 μL
<dual tRNA–sgRNA expression vector (8.0 μL)>
  Golden Gate product 8.0 μL
  10 × Buffer G^∗^ 0.9 μL
  BpiI 0.2 μL
  ***Note***: ^∗^When Esp3I is used in the Golden Gate reaction, Esp3I and 10 × CutSmart buffer are used instead.
(7) Incubate the mixture at 37°C for 60 min, followed by 80°C for 5 min.
(8) For transforming the products into chemically competent cells using a standard protocol, spread the transformation mix on an LB agar plate containing ampicillin.

#### Confirmation of Successful Cloning by Using Colony PCR

(9) Pick a single colony and dissolve in 10 μL of autoclaved milli-Q water. A total of 4–12 colonies are selected to test cloning.
  ***Note***: The correct clones are picked and inoculated into LB medium for DNA preparation.
(10) Heat the solution at 95°C for 5 min and use 2 μL of this solution as template DNA for the following PCR.
(11) Prepare the following PCR mixture (10 μL).
  5 × One Taq Standard Reaction buffer 2.0 μL
  2.5 mM dNTPs 0.8 μL
  10 μM sense oligo for target 0.3 μL
  10 μM tracr-Rv primer 0.3 μL
  Template DNA 2.0 μL
  One Taq DNA Polymerase 0.05 μL
  Autoclaved milli-Q water 4.55 μL
  ***Note***: Primer sequence of tracr-Rv is shown in Supplementary Table 1. The correct assembly of the dual tRNA–sgRNA vector is confirmed using the following primers: sense oligo for second target and antisense oligo for the first target.
(12) Perform PCR under the following conditions:
  (1) 94°C, 30 s
  (2) 94°C, 12 s
  (3) 55°C, 30 s
  (4) 68°C, 20 s
    Repeat steps (2)– (4), 30 times

(13) Run the amplified DNA on 2% agarose gel to check cloning. A band of ∼120 bp implies successful cloning.
  ***Note***: For the dual tRNA–sgRNA vector, a ∼250 bp band is observed.
(14) For further validation of the insertion, analyze the sequence via Sanger sequencing using a NeoUp primer.
  ***Note***: For vectors that use a cassette other than the neomycin resistance gene, use primers for the appropriate drug resistance gene (Supplementary Table 1). blasticidin; BsrUp, hygromycin; HygUp. For doxycycline-inducible vector, use the following primer: blasticidin; BsrDown, neomycin; NeoDown.

### Transformation of *Dictyostelium* Cells

#### Preparation of Plasmid DNA and *Dictyostelium* Cells

(1) Prepare a high-quality CRISPR/Cas9 plasmid DNA using a commercially available plasmid purification kit. The standard amount of DNA for a transient expression vector is 10 μg per transformation. Whereas, for a stable expression vector, 3 μg of DNA is sufficient.
(2) Culture *Dictyostelium* cells (e.g. AX2, AX3 or any other cell line of interest) in HL5 medium at 22°C to a density of 1.5–4.0 × 10^6^ cells/mL. Healthy cells should be used for further experiments and cultured cells older than 2 weeks should be avoided.

#### Transformation of *Dictyostelium* Cells Through Electroporation

(3) Place 1 mm electroporation cuvettes, 1.5 mL tubes containing an appropriate amount of DNA and H50 buffer on ice. The total volume of DNA should be less than 10 μL.
  ***Note***: For the transformation of tag knock-in and precise point mutation, HDR templates should be added.
(4) Transfer the growing cells into 50 mL tubes and incubate on ice for 10 min.
(5) Pellet the cells by centrifugation at 500*g* for 2 min.
(6) Discard the supernatant and wash the cells with 10 mL of ice-cold H50 buffer. Pellet again the cells by centrifugation at 500*g* for 2 min.
(7) Resuspend the pellet in ice-cold H50 buffer at a density of 5 × 10^7^ cells/mL.
(8) Transfer 100 μL of the cell suspension to a 1.5 mL tube containing CRISPR/Cas9 vector.
(9) Transfer the mixture to the electroporation cuvette and electroporate the cells at 0.75 kV, 25 μF, 2 pulses and 5 s pulse interval.
(10) Place the cuvette on ice for 5 min.
(11) To mix the cells, pipette the cells gently up and down and plate the cells in a petri dish containing 10 mL of HL5. Transfer a few hundred microliters of HL5 from the dish to the cuvette, and collect the cell mixture and return to the petri dish.

#### Selection of Transformants

(12) Allow the cells to recover for 8–16 h after electroporation.
(13) Aspirate HL5 medium and replace it with 10 mL of fresh HL5 medium containing appropriate antibiotics. G418; 10 μg/mL, blasticidin; 10 μg/mL and hygromycin; 30 μg/mL.
(14) To induce the transient expression of CRISPR/Cas9 vector, incubate the cells for another 1–3 days before the cells turn round in shape. To select stable transformants, replace HL5 medium few times a week until the transformants become visible. In this process, the antibiotic concentration is increased to G418 20 μg/mL or hygromycin 50 μg/mL.
  ***Note***: A crucial part of transient expression is the selection of appropriate transformants. The duration of antibiotic selection needs to be adjusted according to the condition of cells and the quantity and/or quality of DNA added to the electroporation cuvette. If it is difficult to determine the optimal duration, we recommend that some of the cells from the selection on day 1 be transferred to an SM agar plate as described below and the remainder be maintained on HL5 medium for further antibiotic selection. The resulting transformants can be used directly for the isolation of single clones on SM plates, or the cells can be recovered after a few days of incubation in HL5 medium without antibiotic selection because they contain dead cells.

### Validation of Genome Editing

To identify the desired mutants, perform PCR to amplify the mutated region and sequencing analysis to further validate the desired mutants.

#### Isolation of Single Clones

(1) Collect cells from the transformation plate, count them using a hemocytometer, and the density adjust to 1.5 × 10^4^ cells/mL in KK2.
(2) Plate 100, 35, 15 and 5 μL of the cell mixture containing 1,500, 525, 225 and 75 cells on SM agar plate containing 200 μL of pre-cultured *K. pneumoniae* KpGe strain. Spread the cells over the entire SM plate.
  ***Note***: When cells are plated immediately after transient selection, 75–1,500 cells are inoculated on the plate because they contain dead cells. If rescued cells cultured in HL5 medium for a few days are used, 50–300 cells are transferred to the plate. Certain mutants grow poorly on bacteria, in which case single clones can also be isolated by limiting dilution into 96-well plates containing HL5 medium.
(3) Allow the plates to dry and incubate them at 22°C for ∼4 days. Well-isolated plaque forming colonies will appear on the bacterial lawn of the SM plate containing either diluted cells.

#### Isolation of Genomic DNA for PCR

(4) After individual colonies attain a size of 2 mm, pick single clones with sterile pipette tips and transfer them to PCR tubes containing 20 μL of DNA extraction buffer.
  ***Note***: To save colonies for later culture, label the colonies that were picked from the bacterial plate. The correct clones are picked and inoculated into a 24-well plate or bacterial lawn.
(5) Incubate the mixture at 56°C for 45 min, followed by 95°C for 10 min to inactivate the ProK. This mixture is used as a template for PCR.

#### Genomic PCR for Validation of Genome Editing

(6) Prepare the following PCR mix (10 μL).
  10 × PCR Buffer for KOD -Plus- Neo 1.0 μL
  2 mM dNTPs 1.0 μL
  10 μM sense oligo for target 0.3 μL
  10 μM screening primer Rv 0.3 μL
  Extracted genomic DNA 2.0 μL
  KOD -Plus- Neo 0.2 μL
  Autoclaved milli-Q water 5.2 μL
  ***Note***: A screening primer is designed to amplify the editing region and is used in combination with a sense oligo for target (Figure 3A).
(7) Perform PCR under the following conditions:
  (1) 94°C, 30 s
  (2) 94°C, 12 s
  (3) 58–65°C, 30 s
  (4) 68°C, 20 s
    Repeat steps (2)– (4), 30 times
    ***Note***: For successful mutation–detective PCR, an appropriate annealing temperature should be optimized using the wild-type genome. Because one of the primers, sense oligo for target, is designed to span the mutated nucleotides (Figure 3A), no or weak genome amplification is observed in most of the mutants on performing PCR. PCR of a wild-type genome should be performed as a control. To minimize false-positive amplification, use of a high-fidelity DNA polymerase is recommended.

(8) Validate the mutation using agarose gel electrophoresis. Clones with no or weak amplification are selected as candidates for mutants.

#### Validation of the Mutation Using Sequencing Analysis

Genomic sequencing of the mutated region should be performed for any mutants used for further phenotypic analysis.

(9) Prepare a set of primers to amplify the mutated region.
(10) Perform PCR to amplify the region using the standard protocol.
(11) Run the amplified DNA on agarose gel and purify the desired product using a gel purification kit according to the manufacturer’s instructions.
(12) Validate the mutation using Sanger sequencing.
  ***Note***: For knock-out, indel mutation is observed around three nucleotides upstream of the PAM sequence. In insertions or deletions of three or six nucleotides, no frameshift has occurred and the gene is not entirely disrupted. Mutants with a frameshift are used for further phenotypic analysis. For knock-in and point mutation, ensure that there are no unexpected frameshifts around the target sgRNA or homology arm. For gene deletion, sequencing analysis is unessential if long deletion is observed via PCR. For knock-down, no sequencing analysis is required because dCas9 that lacks nuclease activity is used.

## Expected Results

We used the above procedures to generate a CRISPR/Cas9-mediated gene manipulation system in *Dictyostelium*. An all-in-one vector containing Cas9 and sgRNA was transiently expressed, and several thousands of cells were obtained, of which a portion were used for further screening. The mutants with indel mutations were generated with high efficiency, generally >50% but this ratio differs depending on the target genes. Most of our CRISPR vectors contain a G418-resistant gene. Genome editing within G418-resistant mutants requires vectors with different drug resistance genes. We tested a CRISPR vector with a hygromycin-resistance gene and observed a highly efficient loss of fluorescence resulting from genome editing within the gene encoding for tdTomato (Supplementary Figure 1). The most common method to detect indel mutations is PCR amplification of the targeting region (Figure 3A). Data presented here are a representative result, demonstrating that no PCR band was observed in most of the CRISPR mutants because one of the PCR primers was designed to span the cleavage site (Figure 3B). Even if the mutation was predicted by mutation–detective PCR, it sometimes contained false-positive clones or mutated clones in which multiples of three nucleotides were inserted or deleted. Hence, indel mutations were verified through Sanger sequencing and frameshift mutants were used for further functional analysis (Figure 3B). One of the disadvantages of this method is false-positive clones, where no PCR band is detected, even in non-mutated clones. Hence, long deletions mediated by Cas9 nickase are a straightforward method to detect the mutations effectively because PCR products of the wild-type and mutant genes differ in size (Figures 3C,D). All of the PCR-positive clones exhibited long deletion of the target (Figure 3D). Efficiency of the knockout mutation method was slightly lower (10–30%) than the conventional Cas9 method (>50%), but it was efficient enough to obtain knockout mutants via deletion.

**FIGURE 4:**
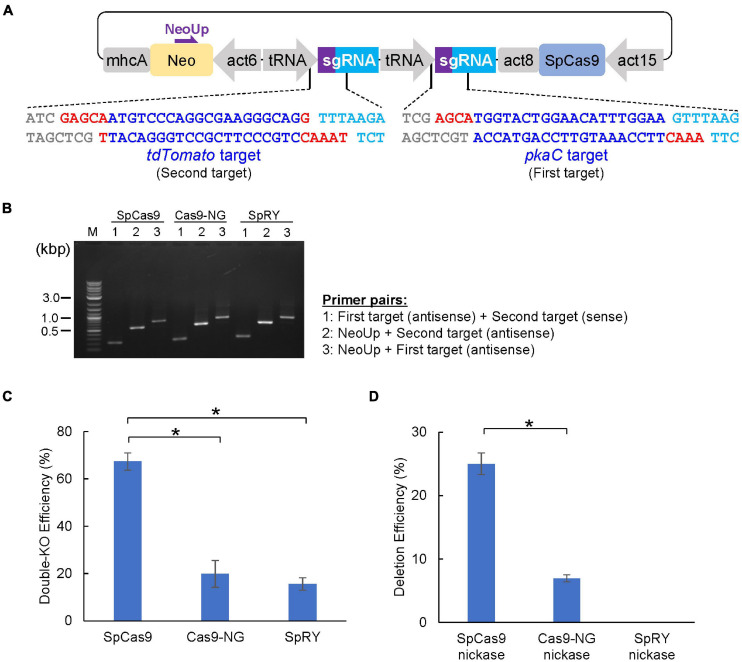
CRISPR/Cas9-mediated double-knockout and genomic deletion. **(A)** Schematic of the dual sgRNA expression vector designed for single cloning step. Red and blue letters represent overhands for the cloning and 20-nt of target sequences, respectively. **(B)** Correct insertion of the first and second targets was confirmed via colony PCR. **(C)** Summary of double knock-out efficiencies by various Cas9 and sgRNA that target *pkaC* and *tdTomato* gene. **(D)** Summary of deletion efficiency in *pkaC* gene by various Cas9 nickase. Data shown are mean ± SEM (*n* = 3 biological replication). *P<0.05.

We constructed a CRISPR/Cas9 vector that disrupts multiple genes simultaneously and successfully modified five PI3K genes (Sekine et al., 2018). However, three steps of Golden Gate cloning were required to produce the targeting vector, which was time consuming. In general, even when analyzing several genes simultaneously, a few genes are often disrupted at the same time. We therefore constructed CRISPR/Cas9 vectors containing two tRNA–sgRNA cassettes to be able to generate a targeting vector for two genes in a single cloning step. We designed a target sequence for both the *pkaC* and *tdTomato* genes, and two pairs of sgRNAs containing different overhangs at the 5′ end of the oligonucleotides were assembled into a CRISPR vector, pTM1725 (Figure 4A). Colony PCR confirmed that the colonies analyzed contained both sgRNA sequences (Figure 4B). The CRISPR vectors were introduced into tdTomato–knock-in cells to determine the ratio of cells in which the two genes were simultaneously disrupted, which would result in loss of red fluorescence and aggregation defects. As a result, both genes were disrupted in over 60% of the mutants with the SpCas9 vector. By contrast, knockouts of both genes using SpCas9-NG or SpRY were less efficient than what we observed for SpCas9 (Figure 4C). Therefore, the use of SpCas9 is preferred unless a NGG PAM cannot be identified in the genes of interest. Furthermore, a nickase (D10A mutant) was generated based on SpCas9-NG and SpRY, and we found that using SpCas9 nickase was the most effective method to generate long deletions (Figure 4D). Hence, CRISPR vectors targeting two genes can be constructed using one cloning step, and the genes of interest are disrupted with high efficiency.

**FIGURE 5:**
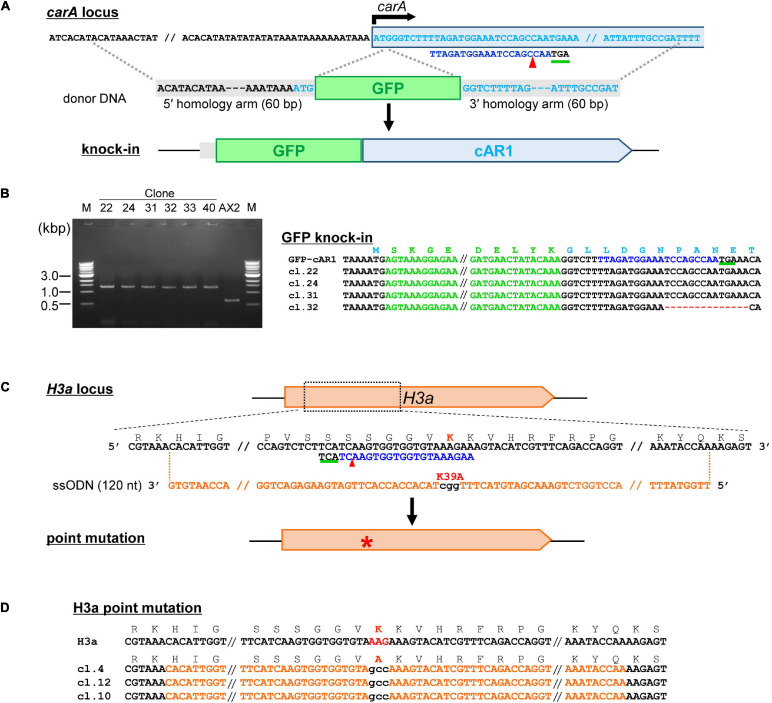
Tagging a protein of interest and precise nucleotide substitutions mediated by CRISPR/Cas9. **(A)** Schematic diagram of tag knock-in of cAR1 locus. Target sequences are in light blue and the PAM sequence is underlined in green. The donor fragment was amplified by PCR using GFP primers flanked with 60-nt homology arms. **(B)** Mutation–detective PCR using primers flanking the knock-in site. The PCR fragments different in size were identified. **(C)** Schematic diagram of precise nucleotide substitutions of H3aK39A. **(D)** Sequencing resutls of mutants for knock-in precise nucleotide substitutions. The target sequence is in blue and mutations are in red. Numbers in parentheses indicate the number of modified nucleotides.

Tagging a protein of interest with an epitope tag or fluorescent protein is one of the most popular methods used to study protein function. Overexpression of the fusion protein enables observation of the protein in cells, but overproduction may lead to artifacts such as ectopic expression or dominant-negative effects. Recent methods based on CRISPR/Cas9-mediated HDR allow for the knock-in of endogenous genes to achieve expression levels close to those observed endogenously. To knock-in GFP immediately after the start codon, we generated a SpRY-based CRISPR vector and donor DNA containing the GFP sequence and homology arms (Figure 5A). About half of the transformed cells exhibited bands ∼700 bp larger than the parental strain when analyzed using mutation–detective PCR (Figure 5B), indicating that knock-in efficiency was high enough. However, when we tried to generate knock-in strains by combining the same donor DNA with other neighboring target sequence, we found that knock-in frequencies were less than 1%. Therefore, trying several target sequences is essential for the successful generation of knock-in strains. CRISPR/Cas9-mediated HDR allows not only for knock-in of tag sequences but also the introduction of precise point mutations in the genome. When we transformed a CRISPR vector with ssODN, which contains substituted nucleotides in the middle of the sequence and 30–80 bp homology arms on each side (Figure 5C), PCR-positive nucleotide substitutions were observed in 6.8–97.7% of the clones. All clones were analyzed by sequencing to confirm correct knock-in and point mutation generation (Figures 5B,D). The use of several target sequences was also effective for obtaining the mutants, because efficiency depended on the position of the target sequence.

**FIGURE 6:**
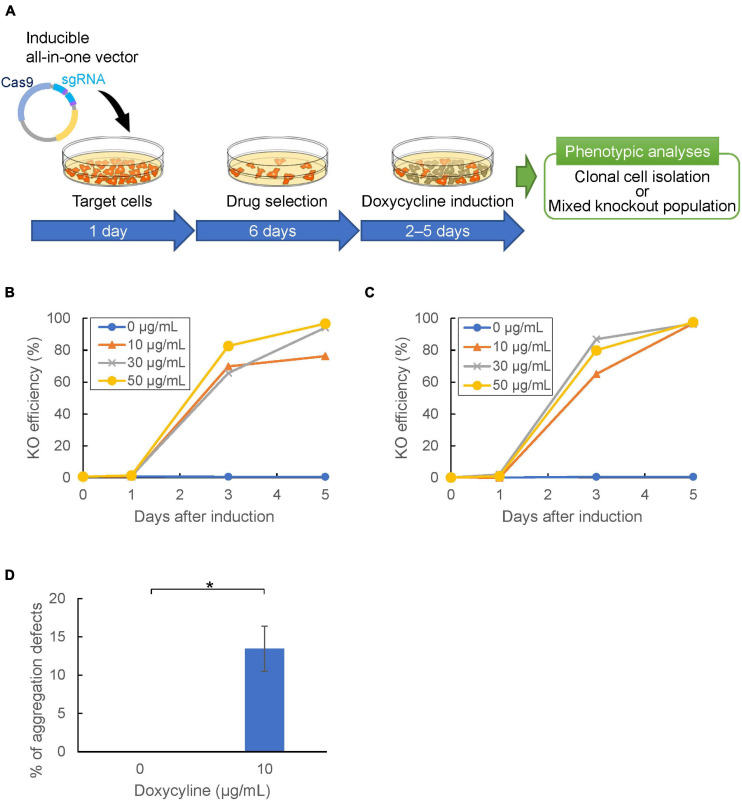
Temporal control of CRISPR/Cas9-mediated gene knockout. **(A)** Gene knockout workflow using inducible all-in-one vector. Target cells were transformed with the inducible vector and selected with Blasticidin or G418 in doxycycline-free HL5 for 6 days. Cas9-stable cells were then incubated in HL5 with doxycycline to induce Cas9. **(B,C)** Temporal control of genome editing via inducible Cas9 vector containing neomycin **(B)** or blasticidin **(C)** resistance cassette, respectively. The mean of knock-out efficiency at different doxycycline concentrations (0, 10, 30, 50 μg/mL) was represented. **(D)** Frequency of aggregation defect mutants targeting *pkaC* gene at different doxycycline concentrations. The error bar shows the standard deviation based on four independent biological replicates. *P<0.001.

Genome editing using CRISPR/Cas9 relies on the nuclease activity of Cas9. This limits its use in applications in which genetic perturbation needs to be controlled temporally; i.e., when it is desired to edit genes that induce strong side effects on growth or development. A drug-inducible CRISPR/Cas9 system allows for temporal control of genome editing. A doxycycline-inducible expression vector was constructed and high efficiency of expression induction was demonstrated in *Dictyostelium* (Veltman et al., 2009b). Therefore, we designed a CRISPR/Cas9 system that enables temporal control of the Cas9 nuclease through drug-treatment. We integrated the target sequence of *tdTomato* into a tRNA–sgRNA cassette of the CRISPR vector to obtain stable expression strains. The loss of fluorescence was then determined with/without doxycycline-treatment (Figure 6A). As a result, loss of fluorescence was observed in more than 60% of cells after 3 days of drug-treatment, whereas no change in fluorescence was observed in cells without doxycycline-treatment (Figures 6B,C). We then selected *pkaC* as a target gene because the knockout mutant exhibits a defect in cell aggregation. Upon treatment with doxycycline for 2 days, approximately 15% of the independent clones were aggregation-negative, whereas all the clones exhibited normal aggregation in the mock controls (Figure 6D). Hence, we developed a drug-inducible CRISPR/Cas9 system that exhibits high knockout efficiency upon induction of Cas9 activity.

**FIGURE 7:**
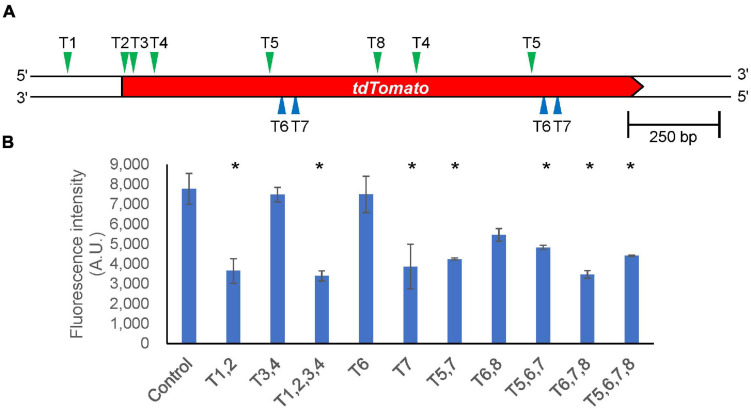
Silencing gene expression by CRISPRi. **(A)** Schematic illustration of target sites in the *tdTomato* knock-in region. The position of each target site on the upper strand and complementary lower strand is indicated by green and blue arrowheads, respectively. **(B)** Intracellular fluorescence intensity in different target combinations. Data shown are mean ± SEM (*n* = 3 biological replication). *P<0.05; ANOVA folloed by Tukey’s *post hoc* test.

In general, it is difficult to analyze the functions of essential genes because their knockout is lethal. Gene silencing that inhibits expression of the target gene is an alternate method to investigate their functions. Although RNA interference (RNAi) has been used for this purpose, CRISPR/Cas9-based gene silencing methods have been developed in various organisms. Catalytically inactive Cas9 (dCas9) lacks endonuclease activity, and it remains bound to specific target DNA sequences, which allows for silencing of gene expression without altering DNA sequences. We designed target sequences for the *tdTomato* gene and co-expressed dCas9 and tRNA–sgRNAs within the tdTomato–knock-in cells. We selected 10 different pairs of target sequences (Supplementary Table 2) and found loss of fluorescence in most of the CRISPRi cell lines. The fluorescence intensity was reduced by about half when the targets were located around the transcription start site (T1,2,3,4) of the gene, but the efficiency of gene silencing varied depending on the targets (Figure 7). We also investigated RNA levels in the CRISPRi cells expressing T1, 2, 3, and 4 sgRNA and found that RNA levels were less than 10% of what was observed for controls. We also investigated whether drug-induced CRISPRi resulted in suppression of fluorescence levels. However, cells in which dCas9 was induced by doxycycline treatment for 4 days exhibited no major repression of either fluorescence or RNA levels. Therefore, constitutive expression of a CRISPRi vector is an effective tool for functional analysis of genes whose knockout produces strong defects in cell growth.

## Discussion

Gene knockout was performed by introducing a linearized DNA construct containing a drug-resistance gene flanked by homology arms complementary to the target gene locus. This method is still widely used in *Dictyostelium*, but CRISPR/Cas9-mediated gene manipulation is just now emerging. The development and improvement of the CRISPR toolbox provides straightforward procedures to generate knockouts, inducible knockouts, knockdowns, knock-ins, point mutations and deletions without integrating a drug-resistance gene. Therefore, the CRISPR toolbox developed in our study increases the robustness of functional analysis in the biomedical model organism *Dictyostelium*.

In the case of knockout using homologous recombination, it is not possible to obtain knockout mutants for essential genes. By contrast, using our CRISPR/Cas9 method, mutants with insertions or deletions of multiples of three nucleotides are generated even if the gene is essential. Indeed, this method was used to identify essential genes (You et al., 2020). Simultaneous editing of multiple genes is achieved by expressing Cas9 and multiple sgRNAs from a single CRISPR vector (Sekine et al., 2018). This is a huge advantage of the CRISPR-mediated knockout method because Cre-loxP–mediated multiple gene disruption is much more time-consuming. The multiplex CRISPR vector is able to carry up to 20 sgRNAs in a single vector with 4-step cloning, but practically, it is unlikely that it is necessary to disrupt more than five genes at the same time. The new CRISPR vectors presented in this study are able to integrate two target sequences in a single cloning step in a single tube reaction. By changing the overhangs of oligonucleotides at the end of the target, it is possible to create vectors containing three or four sgRNAs in one or two Golden-Gate cloning steps, respectively. This reduces the time required to clone and manipulate multiple genes quickly.

This cloning method, which uses multiplex sgRNAs, is useful for CRISPRi-mediated gene repression targeting multiple positions within a single gene. By varying the number of target sequences, the degree of gene repression can be controlled. For repression of gene expression, RNAi has been the most common method of choice since the early 2000s (Martens et al., 2002; Muramoto et al., 2003). The RNAi usually targets gene regions avoiding the UTRs of an mRNA, whereas CRISPRi targets multiple positions in a promoter or a sequence inside a gene, which enables more efficient and fine tuning of gene repression by adjusting the number of targets. In the future, the doxycycline-inducible CRISPR/Cas9 and CRISPRi systems will help us to analyze genes that exhibit strong defects in growth and development.

The CRISPR/Cas9 system can also be adapted to many applications. Genes can be disrupted upon conditional expression of Cas9 during development. Many promoters that control the expression of specific cell types are known in *Dictyostelium* (Williams, 2006; Zhukovskaya et al., 2006; Yamada et al., 2008; Chen et al., 2017). Hence, it should be possible to study the effects of knockout at specific developmental stages. We replaced the *act15* promoter with an *ecmA* promoter that is expressed in pstA cells and then induced a cell-type specific knockout in the gene encoding for a fluorescent protein. However, no distinct loss of fluorescence was observed in either growing cells or cells in the pstA region of the slug. The presence of fluorescence may be due to the stability of the fluorescent protein, which is stable for at least several hours (Deichsel et al., 1999). Genome-wide knockout libraries are also an attractive application of the CRISPR/Cas9 system (Shalem et al., 2014). Unlike the well-established forward genetic approach of restriction enzyme-mediated integration (REMI) mutagenesis (Kuspa, 2006), CRISPR-mediated screening can be designed genome-wide or for sub-pooled targets such as kinases or transcription factors. Hence, further refinement of related methods could lead to a breakthrough in the understanding of *Dictyostelium* genetics.

The versatile CRISPR/Cas9 toolboxes presented herein expand the number of genes available for manipulation in the biomedical model organism *Dictyostelium*. The rapid pace of improvement makes it an important leap in a new era of gene manipulation. We conclude that our established protocols pave the way for efficient and simple genetic manipulation.

## Data Availability Statement

The original contributions presented in the study are included in the article/Supplementary Material, further inquiries can be directed to the corresponding author/s.

## Author Contributions

TM conceived the original idea and supervised the project and wrote the initial draft of the manuscript. KY and HI performed experiments presented in this manuscript. KY, HI, and TM worked closely to analyze and interpret data. YK co-conceptualized and provided materials of NBRP Nenkin. All authors revised the manuscript, and read and approved the submitted version.

## Conflict of Interest

The authors declare that the research was conducted in the absence of any commercial or financial relationships that could be construed as a potential conflict of interest.

## Publisher’s Note

All claims expressed in this article are solely those of the authors and do not necessarily represent those of their affiliated organizations, or those of the publisher, the editors and the reviewers. Any product that may be evaluated in this article, or claim that may be made by its manufacturer, is not guaranteed or endorsed by the publisher.

## Footnotes

1
http://www.rgenome.net/cas-designer/

2
http://www.rgenome.net/cas-offinder/

3
http://crispor.tefor.net/
