# Supplementary material for: CRISPR Toolbox for Genome Editing in Dictyostelium
Source: Front Cell Dev Biol. 2021 Aug 18;9:721630. doi: 10.3389/fcell.2021.721630 (PMC8416318; doi:10.3389/fcell.2021.721630)
Supplement: Supplementary file 1 [file Data_Sheet_1.PDF]

CRISPR toolbox for genome editing in *Dictyostelium*

Kensuke Yamashita[1†], Hoshie Iriki[1†], Yoichiro Kamimura[2], Tetsuya Muramoto[1*]

[1]Department of Biology, Faculty of Science, Toho University, 2-2-1 Miyama, Funabashi, Chiba, 274-8510 Japan.

[2] Laboratory for Cell Signaling Dynamics, RIKEN, Center for Biosystems Dynamics Research (BDR), Suita, Osaka, 565-0874 Japan

*** Correspondence:**

Tetsuya Muramoto

tetsuya.muramoto@sci.toho-u.ac.jp

† These authors have contributed equally to this work.

Supplementary Table S1. List of PCR primers for screening and sequencing.

Name	Sequence (5'- to -3')
tracr-Rv	AAGCTTAAAAAAAGCACCGACTCGGTGCC
NeoUp	TCCTGCAGTTCATTCAGGGC
BsrUp	TTCGGGTATATTTGAGTGGAATG
HygUp	TATCCACGCCCTCCTACATC
NeoDown	ATCATGGTGGAAAATGGCCG
BsrDown	CCTTGTGGTATGTGTAGGGAGT

Supplementary Table S2. List of target sequences for CRISPRi.

Name	Position	Direction	Sequence (5'- to -3')
T1	850	+	agcaTCTTATAAAAAAAAGCCATT
			aaacAATGGCTTTTTTTTATAAGA
T2	1000	+	gagcaATGGACGGTGAAGATGTTCAG
			taaacTGAACATCTTCACCGTCCATT
T3	1024	+	agcaTCCATAAAAATGGTTAGTAA
			aaacTTACTAACCATTTTTATGGA
T4	1084	+	gagcaCGCATGGAGGGCTCCATGAAG
			taaacTTCATGGAGCCCTCCATGCGT
T5	1411	+	gagcaGGCACCAACTTCCCCCCCGAG
			taaacTCGGGGGGGAAGTTGGTGCCT
T6	1436	-	agcaCATGGTCTTCTTCTGCATTA
			aaacTAATGCAGAAGAAGACCATG
T7	1475	-	agcaGTCGCGGGGGTACAGGCGCT
			aaacAGCGCCTGTACCCCCGCGAC
T8	1702	+	gagcaCTGTTCCTGGGGCATGGCACG
			taaacGTGCCATGCCCCAGGAACAGT

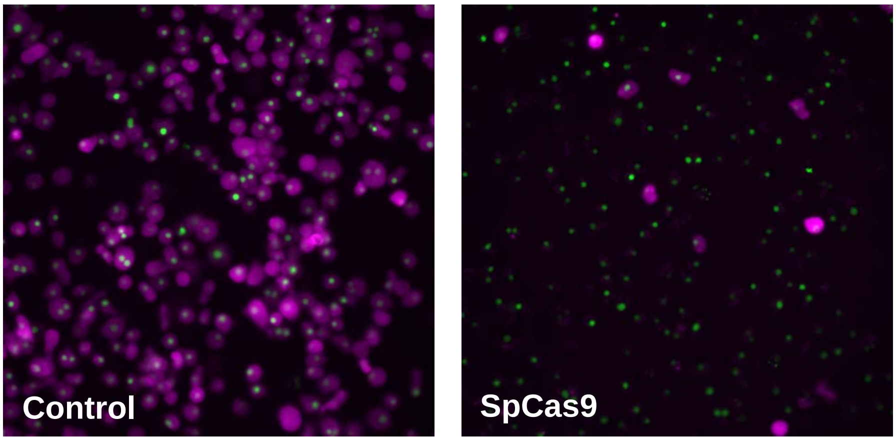

Supplementary Figure S1. Gene mutagenesis by CRISPR/Cas9 vector with hygromycin resistance cassette.
Loss of red fluorescence mediated by the CRISPR/Cas9 all-in-one vector containing hygromycin resistance gene, pTM1756. Control is the tdTomato knock-in cells expressing GFP-H2B as a nuclear marker. The right panel represents cells expressing Cas9 and *tdTomato* sgRNA.
